# NRG Oncology Survey on Practice and Technology Use in SRT and SBRT Delivery****

**DOI:** 10.3389/fonc.2020.602607

**Published:** 2020-11-27

**Authors:** Mikhail Chetvertkov, James Ira Monroe, Jaskaran Boparai, Timothy D. Solberg, Deanna H. Pafundi, Russell L. Ruo, David J. Gladstone, Fang-Fang Yin, Indrin J. Chetty, Stanley Benedict, David S. Followill, Ying Xiao, Jason W. Sohn

**Affiliations:** ^1^ Cancer Institute, Allegheny Health Network, Pittsburgh, PA, United States; ^2^ Department of Radiation Oncology, Mercy Hospital South, St. Louis, MO, United States; ^3^ Operations Department, NRG Oncology, Philadelphia, PA, United States; ^4^ United States Food and Drug Administration, Silver Spring, MD, United States; ^5^ Department of Radiation Oncology, Mayo Clinic, Jacksonville, FL, United States; ^6^ Department of Medical Physics, McGill University Health Centre, Montreal, QC, Canada; ^7^ Radiation Oncology Department, Dartmouth-Hitchcock Medical Center, Lebanon, NH, United States; ^8^ Radiation Oncology Department, Duke University Medical Center, Durham, NC, United States; ^9^ Department of Radiation Oncology, Henry Ford Cancer Institute, Detroit, MI, United States; ^10^ Department of Radiation Oncology, University of California at Davis, Sacramento, CA, United States; ^11^ IROC Houston Quality Assurance Center, University of Texas MD Anderson Cancer Center, Houston, TX, United States; ^12^ Department of Radiation Oncology, University of Pennsylvania, Philadelphia, PA, United States

**Keywords:** stereotactic body radiotherapy, stereotactic radiotherapy, radiotherapy, clinical trials, best practices

## Abstract

**Purpose:**

To assess stereotactic radiotherapy (SRT)/stereotactic body radiotherapy (SBRT) practices by polling clinics participating in multi-institutional clinical trials.

**Methods:**

The NRG Oncology Medical Physics Subcommittee distributed a survey consisting of 23 questions, which covered general technologies, policies, and procedures used in the Radiation Oncology field for the delivery of SRT/SBRT (9 questions), and site-specific questions for brain SRT, lung SBRT, and prostate SBRT (14 questions). Surveys were distributed to 1,996 radiotherapy institutions included on the membership rosters of the five National Clinical Trials Network (NCTN) groups. Patient setup, motion management, target localization, prescriptions, and treatment delivery technique data were reported back by 568 institutions (28%).

**Results:**

97.5% of respondents treat lung SBRT patients, 77.0% perform brain SRT, and 29.1% deliver prostate SBRT. 48.8% of clinics require a physicist present for every fraction of SBRT, 18.5% require a physicist present for the initial SBRT fraction only, and 14.9% require a physicist present for the entire first fraction, including set-up approval for all subsequent fractions. 55.3% require physician approval for all fractions, and 86.7% do not reposition without x-ray imaging. For brain SRT, most institutions (83.9%) use a planning target volume (PTV) margin of 2 mm or less. Lung SBRT PTV margins of 3 mm or more are used in 80.6% of clinics. Volumetric modulated arc therapy (VMAT) is the dominant delivery method in 62.8% of SRT treatments, 70.9% of lung SBRT, and 68.3% of prostate SBRT.

**Conclusion:**

This report characterizes SRT/SBRT practices in radiotherapy clinics participating in clinical trials. Data made available here allows the radiotherapy community to compare their practice with that of other clinics, determine what is achievable, and assess areas for improvement.

## Introduction

Stereotactic radiation therapy (SRT) and stereotactic body radiation therapy (SBRT) are external beam techniques that deliver high doses (2,400 cGy to 6,000 cGy) in a small number of fractions (one fraction, up to five fractions), resulting in a high biological effective dose (BED) to intracranial (SRT) or extracranial (SBRT) targets. The approach is also referred to as Stereotactic Ablative Radiotherapy (SABR). These ablative doses require precise and accurate patient immobilization and positioning, tumor localization, and conformal radiation delivery techniques to allow the use of minimal GTV-to-PTV margins to spare neighboring normal tissues. SRT and SBRT are made possible by several advanced technological developments in radiation therapy, which include the ability to generate high dose beams with steep gradients and direct them with millimeter accuracy using MLC optimization, complex beam fluence shaping, and image guidance. SRT/SBRT delivers high doses in small numbers of fractions to the target volume resulting in a high biological effective dose (BED).

Multiple reports, guidelines, reviews, and textbooks covering recommended best practices of SRT/SBRT have been published, reflecting the growing number of practitioners actively pursuing and expanding this field. Published in 2010, the American Association of Physicists in Medicine Task Group 101 (AAPM TG-101) (1) report provides comprehensive guidance to SBRT treatment delivery as well as recommendations on clinical implementation, quality assurance, quality improvement, and patient safety. Roles and responsibilities of radiation oncologists, medical physicists, and radiation therapists are described in the recommendations of ASTRO/ACR Practice Guidelines for SRS and SBRT (2, 3). The International Commission on Radiation Units and Measurements (ICRU) issued Report 91: Prescribing, Recording, and Reporting of Stereotactic Treatments with Small Photon Beams (4), addressing all aspects of this increasingly important clinical practice. The United Kingdom Stereotactic Ablative Body Radiation Therapy (SABR) Consortium report (5) is a comprehensive overview of the SBRT program recommendations including safety and quality assurance and treatments for the different sites, and Canadian Association of Radiation Oncology scope of practice guidelines (6) is a concise overview of the principles, roles, technologies, practices and safety, and quality assurance in SBRT. More recently, AAPM published practice guideline for SRS, SRT, and SBRT, describing suggested minimum standards for such services (7). And there is an ACR-AAPM technical standard for medical physics performance monitoring of SBRT (8).

Due to the large doses delivered with stereotactic treatments, special attention to the quality assurance and safety aspects of SRT/SBRT program is required as mistakes in any part of the workflow for an SRT/SBRT treatment planning and delivery process could lead to irreversible patient harm. Solberg et al. (9) summarized the quality and safety considerations for a robust and effective SRT/SBRT program.

Guidelines and reports represent guidance for the current clinical best practice in SRT/SBRT and should not be considered as mandatory or regulatory requirements for performing these procedures. As always, the continuing development of technologies and procedures will necessitate continued evolution of existing guidelines in order to encourage best care practices and adapt to technological developments. A key part of this process is to assess how various clinics have implemented and practiced SRT/SBRT and publish the findings for other clinics to reference and compare their own practices.

The purpose of this manuscript is to assess SRT/SBRT practices by polling clinics participating in multi-institutional clinical trials.

## Materials and Methods

The survey was conducted October 2018. It was electronically distributed by the Imaging and Radiation Oncology Core (IROC) Houston Quality Assurance center to the 1,996 radiation therapy institutions that participate in NCTN clinical trials. Participation in the survey was not mandatory. A total of 568 (28.5%) institutions responded to the survey. The survey consisted of 23 questions, which covered general technologies, policies and procedures used in that specific institution for SRT/SBRT delivery (nine questions), as well as site-specific questions for brain SRT, lung SBRT, and prostate SBRT (12 questions for all three sites, one for brain and lung, and one lung-specific). The questions included in the survey are listed in **Table 1** (general SRT/SBRT questions) and in **Table 2** (site-specific questions). To keep the survey concise, other anatomical sites which can be treated with SBRT (*e.g.* head and neck, spine, liver, pancreas, *etc.*) were not included in the survey since SBRT delivery techniques to the brain, lung, and prostate cover a majority of the technical challenges inherent to other anatomical sites (*e.g.* immobilization, positioning/repositioning, motion management, patient monitoring, *etc.*).

**Table 1:** General SRT/SBRT survey questions.

General questions
G1. Does your clinic use a special physics consultation form for SBRT?
G2. What is your policy regarding the physicist’s presence at treatment machine during SRT/SBRT treatment delivery?
G3. Is an attending physician (MD not in training) approval for patient positioning at the machine normally required?
G4. Do you use couch indexing and patient marks for SBRT treatments?
G5. Do you use a threshold for repositioning after On-Board Imaging (OBI)?
G6. If yes, what threshold for repositioning is used?
G7. Do you take a shift verification image after re-positioning? What threshold do you use for taking the verification image?
G8. Do you reposition using non-x ray positioning such as Optical Surface imaging?
G9. If you like to make a change in the SBRT process, what will it be?

**Table 2:** Site-specific SRT/SBRT survey questions.

Site-specific questions (Brain, Lung, Prostate SBRT)
S1. Does your clinic deliver SRT/SBRT to the Brain/Lung/Prostate?
S2. Which immobilization device is generally used?
S3. What size (in mm) of margin is generally used for the PTV?
S4. Which treatment modality is used most often?
S5. What beam arrangement is generally used?
S6. What single energy is generally used most often?
S7. What is the typical maximum dose relative to the prescription?
S8. Which imaging system do you use for the main position correction/verification?
S9. After repositioning do you take a verification image? What imaging technique do you use to verify position?
S10. How do you correct initial setup position?
S11. Do you use a real-time patient monitoring system?
S12. What real-time patient monitoring system do you use?
S13. Brain, Lung SBRT: Do you normally take an extra CBCT (or other images) in order to verify the position in the middle of a treatment fraction?
S14. Lung SBRT: Which motion management system do you use?

Question G1 evaluates how many institutions use a special physics consult form, which is a part of the documentation process and in some instances, necessary for insurance reimbursement and audits.

Questions G2 and G3 identify policies which institutions follow when performing SRT/SBRT procedures, namely the policy regarding the physicist’s presence at the treatment machine during patient positioning and treatment delivery and the policy regarding attending physician image approval for patient positioning before the treatment.

Question G4 evaluates whether couch indexing is used for patient immobilization and setup before SRT/SBRT treatment. Question G5 assesses whether institutions use a shift tolerance for patient repositioning after image acquisition using On-Board Imaging (OBI), and Question G6 reveals those tolerance levels used for patient repositioning. Question G7 determines if a shift verification image is taken after patient repositioning and what threshold, in mm, is generally used to trigger this re-imaging.

Question G8 quantifies how many institutions reposition patients using non-x-ray Surface Guided Radiation Therapy (SGRT) systems.

Finally, question G9 provides a platform for the institution to list the changes they would like to make in their institution’s SRT/SBRT program. This question is a free form and covers both technical [*i.e.*, adding/changing/modifying the technology and techniques, quality assurance (QA) and quality control (QC) tests] and administrative (*i.e.*, changing staffing requirements, documentation process, protocols, *etc*.) issues.

### Site-Specific Questions

Treatment site-specific questions cover brain SRT, lung SBRT, and prostate SBRT programs. Question S1 assesses the number of institutions which perform that specific type of the SRT/SBRT. If that institution does not deliver SRT/SBRT to that specific anatomical site, all subsequent questions for this site are skipped.

Question S2 determines what kind of immobilization devices are used for the specific site to deliver SRT/SBRT treatment. Question S3 evaluates the margin size used for the planning target volume (PTV) expansion. Questions S4 and S5 identify which treatment modality/technique and what beam arrangement is used most often for that SRT/SBRT treatment.

Question S6 evaluates the energy used most often when treating that site at the institution. The choices for this question assume that most institutions use medical linear accelerators to deliver patient treatments; but for those who use other modalities to deliver these treatments, a means to enter their own value and/or comment is presented.

Question S7 asks for the typical maximum dose relative to the prescription allowed. SRT/SBRT treatments are inherently more heterogeneous within the target volumes due to the importance of sharp dose fall-off immediately outside the target area, so question S7 tries to assess the variation in these characteristics among institutions.

Because the use of image guidance for target localization is a prerequisite for accurate SRT/SBRT delivery, questions S8 and S9 document what types of imaging systems are used for target localization as along with the methodology for position correction/verification.

Question S10 evaluates how many degrees of freedom are used by each institution in order to correct the initial patient position relative to the treatment machine. Questions S11 and S12 assess how many institutions use real-time patient monitoring system during the treatment delivery and what kind of monitoring systems are used most often.

Question S13 covers brain SRT and lung SBRT and assesses whether additional verification images are taken during treatment delivery in order to verify patient and/or target positioning.

Question S14 is a lung SBRT specific question and documents which motion management system is used in order to account for or reduce a patient’s respiratory motion.

## Results

Results below show the percentage of participants who responded to each particular question/answer. Survey results were equally weighted, without considering the number of patients being treated or number of procedures performed at each responding institution.

### General Questions

**G1: Does your clinic use a special physics consultation form for SBRT?**

Question G1 revealed that a little over half of the participants (55.3%) who perform SRT/SBRT treatments use a special physics consultation (SPC) form.

**G2. What is your policy regarding the physicist’s presence at treatment machine during SRT/SBRT treatment delivery?**

The AAPM TG-101 report recommends that at least one qualified physicist should be present at the machine for the entire first fraction and be available for the subsequent fractions, particularly for the patient setup “to verify immobilization, imaging, registration, gating, and setup correction” (1).

Almost half (48.8%) of the respondents report the physicist is required to be present at the machine for every fraction of SRT/SBRT. A total of 18.5% report that a physicist’s presence is required for the initial SRT/SBRT fraction only; 14.9% report the requirement of a physicist to be present for the entire first fraction of SRT/SBRT and for the setup approval (but not delivery) for all subsequent treatments. 8.9% of responding clinics state a physicist’s presence at the machine is not required by their policy, and the remaining 8.9% report their policy as “other”.

Institutions that reported “other” (50 respondents) were asked to provide additional details on their institution’s policy. Eighteen out of 50 (3.2%) reported that the physicist’s presence is required at the machine for the patient setup only, but not treatment delivery. Ten out of 50 (1.8%) do not require a physicist’s presence at the machine for the SRT/SBRT patient setup or delivery, but do require a physicist to be readily available in the department if needed. Nine out of 50 (1.6%) reported that a physicist’s presence is required for the initial SRT/SBRT fraction only and to be readily available for all subsequent fractions. The remaining thirteen out of 50 (2.3%) respondents had varying policies regarding physicist’s presence at the machine. These were either site-specific (*i.e.*, the body SBRT policy was different from the cranial SRT) or technique-specific (*i.e.*, whether it is gated, uses breath-hold, Calypso^®^ beacons are utilized, or CyberKnife^®^ vs. linear accelerator is used).

**G3. Is an attending physician approval for patient positioning at the machine normally required?**

AAPM TG-101 (1) recommends that “radiation oncologist approve the result of the image guidance and verify the portal films before every fraction of the SBRT treatment,” while the ACR-ASTRO practice guideline requires that the physician “…attend and direct the actual treatment process.” (3)

For question G3, 55.3% of institutions report that a physician’s approval of patient positioning is required for every fraction, while 22.0% report that physician approval is required only for the patient setup for every treatment fraction, after which the physician should be immediately available in the clinic area. 9.7% require physician presence for the patient setup for every treatment fraction, and then he/she can leave clinic area; 7.2% require physician’s presence at the machine for the patient setup at the first treatment fraction only but being in clinical area to respond if needed. 5.8% responded that physician presence at the machine is not required by their policy.

**G4. Do you use couch indexing and patient marks for SBRT treatments?**

The majority of survey respondents (84.1%) report that patient marks and couch indexing are used for SBRT treatments; 4.8% report that couch indexing without patient marks is used, and the remaining 11.1% use neither couch indexing nor patient marks.

AAPM TG-101 (1) states “body frames and associated fiducial systems may be used for immobilization and coarse localization; however, they shall not be used as a sole localization technique”. The use of couch indexing and patient marks is still helpful for the initial patient setup and can speed up the overall process of patient positioning and target localization. Ultimately, some form of accurate image guidance is required for final localization.

**G5.Do you use a threshold for repositioning after On Board Imaging (OBI)?**

Approximately half of the reporting institutions (49.5%) use a threshold for repositioning the patient after On-Board Imaging, while 50.5% have no threshold defined.

AAPM TG-101 (1) recommends that “action levels should be defined for the residual target positions and patient rotations which, if exceeded, should trigger repositioning of the patient”.

**G6. What threshold for repositioning is used?**

Question G6 builds upon the previous question to quantify threshold levels used by the institutions for patient repositioning after imaging. The threshold is a maximum allowable deviation of the target position, which, if exceeded, requires further patient repositioning before one can proceed with the treatment. A majority of respondents (85.1%) use a threshold of 2 mm or less: 30.5% use a 2 mm threshold, 52.9% use a 1 mm threshold, and 1.7% indicated a 0 mm, or no tolerance for the residual target error which in reality is unattainable due to the inherent uncertainties associated with patient localization. 10.7% of respondents report using a threshold values of 3 to 5 mm, while 4.2% report using a threshold values of more than 5mm. According to AAPM TG-101 (1) action level for repositioning are “likely to be less than the various treatment margins defined for the treatment, and may vary according to institution, equipment, technique, and treatment site”. For certain anatomical sites, *i.e*. brain, threshold of more than 3 mm seems clinically unacceptable, while it is possible to have such threshold for other sites, *i.e.* lung.

**G7. Do you take a shift verification image after re-positioning? What threshold do you use for taking the verification image?**

After the patient is positioned on the table and then moved to the intended treatment position using a dedicated image-guided technique, one can acquire additional imaging data in order to verify that patient’s shift is performed correctly, and the target is within treatment margins. Question G7 asks whether post-shift verification imaging is performed, and if so, what threshold is used.

A total of 29.3% report their institution generally does not acquire post-shift verification images, 38.9% always acquire such images, while 2.7, 10.0, and 9.1% acquire these images for shifts larger than 1, 3, and 5 mm, respectively.

**G8. Do you reposition using non-x-ray positioning such as optical surface imaging?**

A majority of the survey respondents (86.7%) report that they don’t use non-x-ray devices, such as optical surface imaging, for patient repositioning; the remaining 13.3% report using such devices for patient repositioning.

Optical surface imaging devices can be helpful in the initial setup of the patient, and in monitoring patient position during treatment for repositioning if needed.

**G9. If you like to make a change in the SBRT process, what will it be?**

A total of 304 (some of them are remote facilities of a main facility) answers from 253 institutions were gathered from question G9 regarding desired changes to their current SRT/SBRT program:

- A total of 78 (25.7%) would like to add real time tracking/monitoring systems to their current SRT/SBRT program. Examples include: optical surface Imaging systems (*i.e.* VisionRT AlignRT^®^, Varian HumediQ Surface-Guided Positioning & Monitoring, C-Rad Catalyst™), infra-red (IR) monitoring systems (BrainLab ExacTrac^®^ Infrared Monitoring), fiducials/beacons (e.g., Calypso^®^ lung/prostate beacons), real-time MR guidance, x-ray verification (*e.g.*, BrainLab ExacTrac^®^, fluoroscopy, beam-on imaging using OBI), and ultrasound-based systems (10).
- 50 (16.4%) respondents were completely satisfied with their current SRT/SBRT program implementation and claimed that no changes were required at the moment.
- 42 (13.8%) respondents report they would like to add/update respiratory motion management systems. Examples include breath-hold (Elekta active breath control, DynR SDX), gating [Varian Real-time Position Management™ (RPM) Respiratory Gating], abdominal compression, and 4D Cone-Beam CT (4D-CBCT).
- 42 (13.8%) report they would like to update their documentation, protocols, or procedures in their current SRT/SBRT program implementation such as their institution policies regarding the presence of the physicist or physician at the machine during the SRT/SBRT delivery, more robust patient selection process for the SBRT treatments, *etc.*
- 22 (7.2%) would like to add a six degree-of-freedom couch for the better patient repositioning.
- 21 (6.9%) would like to change or update their current equipment (*i.e.* upgrade linear accelerators, imaging systems, 4D-CT Simulator), software (*i.e.* for the more efficient image registration), or add new technologies (*i.e.* online adaptive re-planning).
- 16 (5.3%) want to update or improve patient immobilization technologies or techniques to make them easier to use, more time-efficient, and reusable.
- 16 (5.3%) would like to add flattening filter free (FFF) beams to their institution.
- The remaining 5.6% listed miscellaneous desired changes to their current program, such as trying different planning techniques (single-iso multiple metastasis, non-coplanar beams, Varian HyperArc™), implementing other delivery techniques to reduce treatment times (*i.e.* arc treatments), adding new treatment sites to their current program, adding new imaging systems (*i.e.* BrainLab ExacTrac^®^), updating their current dose calculation algorithm.

### Site-Specific Questions

**S1. Does your clinic deliver SRT/SBRT to the Brain/Lung/Prostate?**

Question S1 assesses which anatomical sites are treated by the responding clinics. 77.0% of respondents report they perform cranial SRT treatments, 97.5% treat lung lesions with SBRT, and 29.1% of respondents treat prostate with SBRT.

**S2. Brain/Lung/Prostate SRT/SBRT: Which immobilization device is generally used?**

#### Brain SRT Immobilization

For question S2, physicists were asked to describe what kind of immobilization devices are generally used in their institutions, depending on the treatment site. For brain SRT, 57.0% report using a closed head mask down to chin, 13.1% use an extended mask down to shoulder, 20.4% use an open mask, and 9.5% reported using ‘other’ types of immobilization.

‘Other types of immobilization’ was generally used by respondents to describe the use of a combination of different devices, *i.e.* head mask along with bite block and foam down to shoulder, BrainLab, Klarity^®^, Macromedics^®^, or Encompass™ stereotactic solutions, Aktina Pinpoint system, TrUpoint ARCH™ SRT Immobilization System, Fraxion bite-block, *etc.* Several of the respondents who chose ‘other’ use a Gamma-Knife for brain SRT. Generally this implies they use a stereotactic frame for immobilization and localization purposes, although the GammaKnife ICON does permit mask immobilization together with image guided localization.

#### Lung SBRT Immobilization

For immobilization of lung SBRT patients, 36.5% of respondents use vacuum bags, 24.6% use a body vacuum system, 21.8% use a compression device, 10.3% use ‘other’ types of immobilization, and 6.9% report they are not using special immobilization devices.

A majority (40 out of 51) of the institutions that chose the answer ‘other’ use a combination of a vacuum bag and a compression device, *i.e.*, systems such as CIVCO Vac-Lok, QFix, Elekta BodyFix. Eight out of 51 use a specific lung SBRT immobilization system which has immobilization, patient support, and abdominal compression integrated into one platform. These include the CIVCO Body Pro-Lok™ and the Orfit SBRT solution with Vac-Loc. 3 out of 51 report using foam Alpha Cradles.

#### Prostate SBRT Immobilization

For the prostate SBRT, 66.2% of respondents use vacuum systems on a flat couch for patient immobilization, 5.6% use ‘other’ types of immobilization, and 28.2% use no immobilization. Five out of eight institutions that chose the answer “other” use knee and leg immobilization and support, while three report using more advanced patient immobilization and support systems such as the CIVCO Combifix™, CIVCO Pro-Lok™ and Orfit Pelvicast™.

**S3. What size of margin (in mm) is generally used for the PTV?**

For brain SRT, a majority of the institutions (83.9%) use a PTV margin of 2 mm or less: 3.5% use a 0 mm margin for the PTV, 44.0% use a 1 mm margin, and 36.4% use a 2 mm. The remaining institutions report using a 3 mm PTV margin (13.8%) and 2.3% use 4 mm or greater PTV margin.

For lung SBRT the majority of institutions (80.6%) report using a PTV margin of 3 mm or more: 25.9% use 3 mm, while 54.7% use 3 mm or greater margin. 14.5% of institutions use a 2 mm PTV margin, while 4.9% use a 1 mm margin.

Finally, for prostate SBRT, a majority of the institutions (84.5%) report using a PTV margin of 3 mm or more: 42.3% use 3 mm, while 42.2% use 4 mm or greater margin. The remaining institutions report use of a 2 mm PTV margin (12.0%) and a 1 mm margin (3.5%). All of the responses are summarized in **Table 3**.

**Table 3:** Margin (mm) generally used for the PTV.

PTV Margin	Prostate	Lung	Brain
4 mm or greater	42.2%	54.7%	2.3%
3 mm	42.3%	25.9%	13.8%
2 mm	12.0%	14.5%	36.4%
1 mm	3.5%	4.9%	44.0%
0 mm	0.0%	0.0%	3.5%

**S4. Which treatment modality is used most often?**

VMAT/RapidArc is the technique used by a majority of institutions to deliver their SRT/SBRT treatments. This treatment technique is used by 62.8% of institutions performing brain SRT treatments, 71.5% of institutions for lung SBRT, and 69.3% of institutions for prostate SBRT.

For brain SRT, other modalities reported include static gantry IMRT (5.0%), circular cones (4.0%), dynamic conformal arc (14.3%), CyberKnife^®^ (11.1%), and Gamma-Knife (2.8%). For lung SBRT, other modalities reported include static gantry IMRT (9.4%), dynamic conformal arc treatment (11.2%), CyberKnife^®^ (7.3%), and ‘other’ (0.6%, three Tomotherapy institutions). Prostate SBRT modalities other than VMAT include static gantry IMRT (3.6%), dynamic conformal arc (0.7%), CyberKnife^®^ (22.1%), ViewRay (1.4%), and other (2.9%, three Tomotherapy and one proton therapy). All of the responses are summarized in **Table 4**.

**Table 4:** Treatment modality used most often.

Devices/Modalities	Prostate	Lung	Brain
Other	2.9%	0.6%	0.0%
ViewRay	1.4%	0.0%	0.0%
Gamma Knife	0.0%	0.0%	2.8%
CyberKnife	22.1%	7.3%	11.1%
Dynamic conformal arc	0.7%	11.2%	14.3%
Linac with Cones	0.0%	0.0%	4.0%
IMRT	3.6%	9.4%	5.0%
VMAT/RapidArc	69.3%	71.5%	62.8%

**S5. What beam arrangement is generally used?**

For brain SRT, the use of non-coplanar beam arrangements was reported by most responders (79.7%), while the remaining (20.3%) use coplanar beams. In contrast, coplanar beams are used in most lung and prostate SBRT cases (66.9 and 71.1%, respectively).

**S6. What single energy is generally used most often?**

Clinics reported using primarily 6 MV energy beams with and without flattening filters: 50.0, 53.8, and 45.3% using 6 MV, and 40.7, 38.6, and 28.8% using 6 MV FFF, in the brain, lung and prostate, respectively.

For brain SRT treatments, energies other than 6 MV reported were: 10 MV FFF photons (7.3%) and ^60^Co for Gamma-Knife units (2.0%). For lung SBRT, other energies used most often included 10 MV (1.2%) and 10 MV FFF (6.4%). According to AAPM TG-101 report (1) energies higher than 6 MV in lung SBRT treatments should be used with caution due to the larger beam penumbra and lateral range of secondary electrons in lower-density medium, such as lung.

For prostate SBRT, other energies reported included 10 MV (3.6%), 10 MV FFF (20.2%), 15 MV or higher (0.7%), and other (1.4%), the latter of which includes the ViewRay ^60^Co unit and protons. A summary of all of the energies used most often for the three anatomical sites is listed in **Table 5**.

**Table 5:** Single energy generally used most often.

	Prostate	Lung	Brain
Co-60 (1.25 MeV)	0.0%	0.0%	2.0%
6 MV	45.3%	53.8%	50.0%
6 MV FFF	28.8%	38.6%	40.7%
10 MV	3.6%	1.2%	0.0%
10 MV FFF	20.2%	6.4%	7.3%
15 MV or higher	0.7%	0.0%	0.0%
Other	1.4%	0.0%	0.0%

**S7. What is the typical maximum dose relative to the prescription?**

For brain SRT, 38.5% of institutions report a maximum dose of less than 120%, 45.7% report 120–130%, 8.3% report 130–140%, 5.0% report 140–150%, and 2.5% report greater than 150% for the maximum dose. For lung SBRT, 42.8% report a maximum dose of less than 120%, 39.0% report 120–130%, 13.8% report 130–140%, 3.6% report 140–150%, and 0.8% report greater than 150% for the maximum dose. Finally, for lung SBRT, 70.4% report a maximum dose less than 120%, 24.0% report 120–130%, 2.8% report 130–140%, 2.1% report 140–150%, while 0.7% report greater than 150% for the maximum dose. A summary of all of the typical maximum doses relative to the prescription for the three anatomical sites is listed in **Table 6**.

**Table 6:** Typical maximum dose relative to the prescription.

Maximum Dose	Prostate	Lung	Brain
>150%	0.7%	0.8%	2.5%
140–150%	2.1%	3.6%	5.0%
130–140%	2.8%	13.8%	8.3%
120–130%	24.0%	39.0%	45.7%
<120%	70.4%	42.8%	38.5%

**S8. Which imaging system do you use for the main position correction/verification?**

For brain SRT, 59.4% of clinics report using CBCT for their main position correction/verification methodology, 16.5% use 2D-kV or portal imaging, 1.2% use a surface tracking system, 1.0% use no imaging, and 21.9% (88 institutions) use “other”. In the survey, it was asked to specify what imaging techniques are used by the institution if they use other systems more than one type. Out of these 88 institutions, 31 use BrainLab ExacTrac^®^, 15 use a combination of CBCT and kV/port images, 11 use CBCT and a surface tracking system, 11 use BrainLab ExacTrac^®^ and CBCT, six use the CyberKnife Synchrony^®^ system with its integrated imaging system. Four institutions use three systems: CBCT, kV, and surface tracking. Four use a combination of BrainLab ExacTrac^®^ and kV images, three use Gamma Knife (one with Icon™ CBCT system), two use the Tomotherapy system with megavoltage CT (MVCT), and one reports the use of kV/port images and surface tracking.

For lung SBRT, 80.2% of responding institutions use CBCT as the primary position correction/verification system, 7.5% use 2D-kV and portal images, 0.2% use a surface tracking system, and 12.1% (60 institutions) use a combination of the different imaging types. Of these 60 respondents, 19 use a 2D-kV/MV and CBCT combination, 10 use a CBCT and surface tracking, eight use BrainLab ExacTrac^®^, seven use the Tomotherapy MVCT system, five use BrainLab ExacTrac^®^ and CBCT, four use 2D-kV, CBCT and fluoroscopy, four use CyberKnife Synchrony^®^ system, and three use 2D-kV, CBCT and surface tracking.

For prostate SBRT treatments, 59.2% of the institutions use CBCT, 23.2% use 2D-kV or port imaging, 2.8% use radio markers (Calypso^®^ RF beacons), and 14.8% (21 institutions) use a combination of the imaging types, including 2D-KV, CBCT, fluoroscopy, ultrasound, surface tracking, MVCT, radio markers, BrainLab ExacTrac^®^ and 0.35 T magnetic resonance (MR) imaging by ViewRay^®^. The summary of all imaging systems used for the three anatomical sites is listed in **Table 7**.

**Table 7:** Which imaging system generally used for the main position correction/verification.

	Prostate	Lung	Brain
CBCT	59.2%	80.2%	59.4%
2D KV or port imaging	23.2%	7.5%	16.5%
Radio marker	2.8%	0.0%	0.0%
Optical surface imaging	0.0%	0.2%	1.2%
No imaging used	0.0%	0.0%	1.0%
Other	14.8%	12.1%	21.9%

**S9. After repositioning do you take a verification image? What imaging technique do you use to verify position?**

The respondents indicated that 70.2% of institutions practicing brain SRT, 95.4% practicing lung SBRT, and 83.8% practicing prostate SBRT report they acquire verification images following patient repositioning.

For brain SRT, 32.9% report use of the CBCT for the position verification, 25.8% use 2D-kV or portal imaging, 0.5% use a surface tracking system for verification, and 11.0% (44 institutions) use a combination of the different imaging types. 25 out of 44 use BrainLab ExacTrac^®^ for position verification, 10 use the combination of CBCT and 2D-kV or portal images, five use CyberKnife Synchrony^®^ imaging system, and the remaining four use MV portal imaging, Tomotherapy MVCT, or fluoroscopy. 29.8% of institutions do not acquire verification images following patient repositioning.

For lung SBRT, 60.9% report the use of CBCT for position verification, 17.6% use 2D-kV or portal imaging, 7.5% use OSMS system, and 9.3% (46 institutions) use imaging only in certain situations, or a combination of the different imaging types. 13 out of 46 respondents report they use imaging for position verification only in certain situations, such as if patient shifts in one direction or the absolute value of displacements are more than preset threshold. Six institutions use Tomotherapy MVCT, six use a combination of CBCT and surface tracking, six use CBCT and 2D-KV images, five use fluoroscopy, four use the CyberKnife Synchrony^®^ imaging system, four use BrainLab ExacTrac^®^, one uses a combination of CBCT and ExacTrac^®^, and one uses CBCT and fluoroscopy. 4.7% institutions report they do not acquire verification images following patient repositioning.

For prostate SBRT, 38.0% report the use of CBCT for position verification, 28.2% use 2D-kV or portal imaging, 3.5% use radio markers (Calypso^®^ RF beacons), 1.4% use a surface tracking system, and 12.7% (18 institutions) use a combination of the different imaging types. For these 18 institutions, combinations may include: CBCT, fluoroscopy, ultrasound, surface tracking, MVCT, radio markers, BrainLab ExacTrac^®^, Calypso^®^ RF beacons, radio-opaque markers, and 0.35 T magnetic resonance (MR) imaging by ViewRay^®^. 16.2% reported they do not acquire verification images following patient repositioning. A summary of the responses regarding the imaging system used for position localization is shown in **Table 8**.

**Table 8:** What imaging technique generally used for position verification.

Post Shift Verification	Prostate	Lung	Brain
CBCT	38.0%	60.9%	32.9%
2D KV or portal imaging	28.2%	17.6%	25.8%
Optical surface imaging	1.4%	7.5%	0.5%
Radio marker	3.5%	0.0%	0.0%
No imaging used	16.2%	4.7%	29.8%
Other	12.7%	9.3%	11.0%

**S10. How do you correct initial setup position?**

For brain SRT, 76.1% of the institutions correct the initial setup position using six degrees of freedom (DOFs—translations plus roll, pitch, and yawn); 15.1% correct three degrees of freedom (translations only); 6.8% correct four degrees of freedom (translations plus roll); 2.0% (nine institutions) report they use different correction techniques. Of these eight institutions, one report using six DOF correction when their patients are treated on a Varian TrueBeam machine, and four DOFs when treated on a Varian Trilogy machine, two report they correct four DOFs (translation and yaw), three institutions use Gamma-Knife and frame-based localization, and two institutions typically correct translation, but if CBCT reveals patient rotation, they would manually reset the patient.

For lung SBRT, 50.7% of the institutions correct six DOFs, 37.6% correct three translations only, 9.9% correct four DOFs (translations plus roll), and 1.8% (nine institutions) correct differently. Seven out of these nine institutions correct four DOFs (translations plus yaw), one correct five DOFs (translations plus yaw and roll), and one uses different correction techniques depending on the machine used for the treatment (three DOFs and six DOFs).

For the prostate SBRT, 69.0% correct six DOFs, 23.2% correct translations only, 7.1% correct four DOFs (translations plus roll), and 0.7% (one institution) corrects translations plus yaw.

**S11. Do you use a real-time patient monitoring system?**

50.3, 37.8, and 49.3% of respondents report they use real-time patient monitoring for brain SRT, lung SBRT, and prostate SBRT, respectively.

**S12. What real-time patient monitoring system do you use?**

For brain SRT, 47.5% report the use of an optical tracking system, 25.2% use an infrared (IR) sensor array, 1.5% use radio markers, and 25.8% use x-ray systems (including BrainLab ExacTrac^®^, or CyberKnife Synchrony^®^).

For lung SBRT, 49.7% use an optical tracking system, 36.8% use IR sensor array, and 13.5% use x-ray systems.

For prostate SBRT, 54.3% use x-ray systems, 20.0% use radio markers (Calypso^®^), 14.3% use an optical tracking, 1.4% use an IR sensor array, and 10.0% (7 institutions) use other mechanisms, such as ultrasound (3 out of 8 institutions), and other 4 institutions use no real-time monitoring.

**S13. Brain SRT, Lung SBRT: Do you normally take an extra CBCT (or other images) in order to verify the position in the middle of a treatment fraction?**

For brain SRT, 35.3% report they normally acquire an extra CBCT, or other image, in order to verify the patient position in the middle of the treatment, 53.3% don’t normally acquire an extra image, and the remaining 11.4% (45 institutions) only acquire an extra image under certain circumstances. Seven out of 45 use an optical tracking system data in order to decide whether additional imaging is required, and 38 institutions report that decision on whether to acquire additional images is situationally dependent, for example:

- At every different couch angle positions;
- Depending on the proximity to the OAR;
- For single fraction SRS multiple arcs;
- For site-specific cases only (spine, lung, *etc*.);
- When there is a physician order;
- Only if patient motion is observed;
- Always for the first fractions, sometimes for subsequent fractions
- If multiple isocenters are used

For lung SBRT, 15.7% report they acquire an extra image in the middle of the treatment, while 84.3% do not.

**S14. Lung SBRT: Which motion management system do you use?**

36.4% of institutions practicing lung SBRT report they use a motion encompassing technique by defining the Internal Target Volume (ITV), for their motion management. 11.3% use gating [by means of Varian RPM™, ANZAI belt, optical tracking, or Elekta Active Breathing Coordinator™ (ABC)], 6.9% use breath hold (same systems as used for the gating), 6.5% use x-rays to track the tumor motion (*i.e.*, BrainLab ExacTrac^®^, CyberKnife Synchrony^®^), 14.0% use abdominal compression, and 24.9% (123 institutions) use some combination of these systems.

A majority (40 out of 123) use a combination of an ITV and motion restriction to manage the tumor motion; 14 use an ITV and breath hold technique. The remaining 59 use different combinations of ITV, motion restriction, breath hold, gating, x-ray tracking, optical tracking, and fiducials for lung tumor motion management. 10 institutions report they use a free breathing CT scan to plan patients, and treat with no motion management. A summary of the responses of which motion management systems are used for Lung SBRT is listed in **Table 9**.

**Table 9:** Lung SBRT: Which motion management system do you use?

Motion Management System	
Motion encompassing (ITV)	36.4%
Gating	11.3%
Breath-Hold	6.9%
Tracking (x-rays)	6.5%
Motion restriction (abdominal compression)	14.0%
Other, or multiple systems	24.9%

## Discussion

Findings of this survey show that current practices for performing SRT/SBRT treatments in different institutions can vary widely and do not always correlate with published recommendations such as the AAPM’s TG-101 report. Even among published guidelines, recommendations can be different. Such results could directly impact the assumption of consistent clinical performance while participating in clinical protocol studies which is critical for outcome comparisons.

For example, question G2 regarding the physicist’s presence at treatment machine during SRT/SBRT treatment delivery has slightly different recommendations. The AAPM and the Radiosurgery Society (AAPM-RSS) MPPG Practice guideline 9.a. gives the detailed recommendations, stating: “for the first treatment session, a qualified medical physicist (QMP) with relevant SRS-SBRT training must provide personal supervision of the entire session. For any subsequent treatment sessions, direct supervision must be provided by either a QMP or a medical physicist who was present during the initial treatment session” (7). These recommendations closely resemble the AAPM TG-101 report recommendations that at least one qualified physicist should be present at the machine for the entire first fraction, and be available for the subsequent fractions, particularly for the patient setup “to verify immobilization, imaging, registration, gating and setup correction” (1). ACR–ASTRO Practice Parameter only notes that supervision by medical physicist should be performed according to institutional guidelines (3) and further referring to ACR-AAPM Technical Standard, saying that a “QMP is responsible for the technical aspects of SBRT and must be available for consultation and supervision throughout the entire SBRT procedure”, and “ensuring that the beam-delivery process on the treatment unit accurately fulfills the prescription of the radiation oncologist” (8). According to the survey results, a total of 32.4% of clinics (18.5% of the clinics, which require physicist’s presence for the initial SRT/SBRT fraction only, 8.9% of the clinics, which do not require physicist’s presence, 3.2% who require physicist’s presence for setup only, and 1.8% who only require direct, but no personal, physicist’s supervision) do not strictly meet the AAPM-RSS MPPG Practice guideline 9.a of requiring a physicist presence (personal supervision) for the first fraction and patient setup of subsequent fractions and direct supervision for the rest of the subsequent fractions.

In contrast, regarding question G3, TG-101 states that “radiation oncologist approve the results of the image guidance and verify the port films before every fraction of the SBRT treatment” (1). ACR–ASTRO Practice Parameter 2019 notes that supervision by radiation oncologist should be performed according to institutional guidelines and adds “The radiation oncologist should approve the image guidance and motion review and be present at the start of each treatment fraction (3). Our survey results show that most clinics follow these recommendations.

For question S2 reports and guidelines are showing consensus on the use of immobilization. Most references agree this is an essential device for SRT/SBRT treatments, helping reduce the motion, improve stability and reproducibility of the patient setup (1, 3, 8). Survey results show nearly all clinics follow this recommendation.

The answers to the question S14 showed that most of the institutions use AAPM Task Group 76 as a reference for the management of respiratory motion (11). The importance of respiratory motion management is also stated in other reports and recommendations (1, 3, 8). TG-101 addresses the use of optical tracking techniques, which is not discussed in TG-76, but is currently a popular choice for motion management and tracking among radiation therapy institutions. The ASTRO model policy on SBRT treatment states: “Thus, reliable immobilization or repositioning systems must often be combined with devices capable of decreasing organ motion or accounting for organ motion—*e.g.*, use of respiratory gating or robotic target tracking for target sites in the chest or upper abdomen” (11). However, question S11 on the specific application of real-time motion management shows only 37% of clinics use this technique with SBRT.

## Conclusion

Results of this survey allow clinics to cross reference their programs and practices with the community at large, letting clinics know if they are falling behind, are ahead, or struggling with the same issues as other clinics and trying to follow the various published protocols, task groups, and guidelines.

This survey also has implications for multi-institutional clinical studies which depend on consistent treatment planning and delivery among participating clinics for study integrity. Based on the variability in interpreting and enforcing treatment guidelines we believe protocol authors should (1) reference a standard to be followed such as the AAPM’s TG-101 for the first treatment fraction and for subsequent treatment sessions, (2) specify training and credential therapists for SBRT setup if RO and/or QMP are not reviewing daily setup images, (3) recommend appropriate imaging technology, and (4) provide a minimal PTV margin appropriate to the imaging technology used for IGRT.

## Data Availability Statement

The raw data supporting the conclusions of this article will be made available by the authors, without undue reservation.

## Author Contributions

MC, JM, TS, DP, RR, DG, F-FY, IC, SB, DF, and JS wrote and proof read the manuscript. JB created the survey. All authors contributed to the article and approved the submitted version.

## Funding

This project was supported by grants U24CA180803 (IROC) and U10CA180868 (NRG Oncology Operations) from the National Cancer Institute (NCI).

## Conflict of Interest

DF reports grants from NCI, during the conduct of the study. IC reports grants from Varian Medical Systems and Philips HealthCare, outside the submitted work.

The remaining authors declare that the research was conducted in the absence of any commercial or financial relationships that could be construed as a potential conflict of interest.
